# Breast Reconstruction after Mastectomy in Women with Breast Cancer: A Systematic and Meta-Analysis Review

**DOI:** 10.29252/wjps.9.1.3

**Published:** 2020-01

**Authors:** Amir Anbiyaiee, Mahrokh Abouali Galeh Dari, Omid Anbiyaee, Abolghasem Anbiyaiee

**Affiliations:** 1 Department of Obstetrics and Gynecology, School of Medicine, Ahwaz Jundishapur University of Medical Sciences, Ahwaz, Iran; 2 Department of Dermatology, School of Medicine, Shiraz University of Medical Sciences, Shiraz, Iran; 3 Radiology Department, Golestan Medical Center, Ahvaz Jundishapur University of Medical Sciences, Ahvaz, Iran

**Keywords:** Breast, Reconstruction, Mastectomy, Cancer

## Abstract

**BACKGROUND:**

Immediate Breast Reconstruction (IBR) is an additional surgical procedure that may increase postoperative complications (such as flap necrosis, infection, and hematoma) and delay the initial time for adjuvant chemotherapy in some patients. In this systematic and meta-analysis, we provide overall survival rates of patients who underwent mastectomy with and without IBR.

**METHODS:**

The following databases were systematically searched between 2015 to 2019 without language restrictions in PUBMED, EMBASE, Web of Science, and Cochrane Library. In addition, the relevant references in the list of all included articles were also checked. The search term included “breast cancer” and “breast reconstruction” “mastectomy”.

**RESULTS:**

The sample size was a range from 339 to 5644 patients. The median age was 46.3 years. The results showed no significant differences in terms of overall survival between two groups.

**CONCLUSION:**

The results showed that IBR after mastectomy did not affect the overall survival.

## INTRODUCTION

Breast cancer is the leading cause of cancer and death in women worldwide.^1^ Its surgical approach has become less and less mutilating in the last decades. However, the overall number of breast reconstructions has significantly increased lately.^2^ Nowadays, breast reconstruction should be individualized at its best, first of all taking into consideration not only the oncological aspects of the tumor, neo-/adjuvant treatment, and genetic predisposition, but also its timing (immediate versus delayed breast reconstruction), as well as the patient’s condition and wish.^3^ Breast cancer is the leading cause of cancer death among women worldwide with ~1.7 million new diagnoses and 521.900 deaths in 2012.^4^^,^^5^

One important modality of breast cancer therapy is surgical treatment, which has become increasingly less mutilating over the last century.^6^^,^^7^ Mastectomy aims at resecting as much breast tissue as possible, knowing that glandular tissue will almost always remain in the region of the inframammary fold.^8^ Breast reconstruction after mastectomy is oncologic ally safe and is associated with high satisfaction and improved psychosocial outcomes.^9^ Although the rates of major complications after immediate reconstruction are greater than after mastectomy alone, clinically significant delays in the receipt of adjuvant therapy after immediate reconstruction have not been found.^10^

Breast reconstruction (BR) is an option for women who are treated with mastectomy; however, there has been concern regarding the oncologic safety of BR.^11^ Breast reconstruction is dependent primarily on the type of mastectomy and may be classified in various ways, such as reconstruction type and reconstruction time point. Breast reconstruction is a surgical procedure that restores shape to breast after mastectomy surgery that removes breast to treat or prevent breast cancer.^12^

Breast reconstruction surgery aims to restore the appearance of a natural breast after a mastectomy and can help a woman look and feel better. Many women find that breast reconstruction significantly improves their self-image, self-confidence and quality of life. On the practical side, breast reconstruction eliminates the need for external artificial prostheses, which can sometimes be uncomfortable to wear.^13^ The latter includes delayed breast reconstruction (DBR; secondary breast reconstruction) and immediate breast reconstruction during the same surgery (IBR; primary breast reconstruction).^2^

IBR is advantageous over DBR, because it decreased the total number of surgical procedures and the risks therein. Breast reconstruction surgery is the creation of a new breast shape, or mound, using surgery. IBR is an additional surgical procedure, it may increase postoperative complications (such as flap necrosis, infection, and hematoma) and delay the initial time to adjuvant chemotherapy in some patients.^14^ It may be done after removal of a whole breast (mastectomy) or part of the breast (breast-conserving surgery).^15^ Nonetheless, IBR is advantageously associated with a reduced recovery time, a better esthetic outcome, an improved quality of life, lower surgery, and recovery related costs.^12^^,^^16^^,^^17^ In this systematic and meta-analysis, we provided overall survival between patients who underwent IBR after mastectomy.

## MATERIALS AND METHODS

*Search Strategy*

**Fig. 1:**
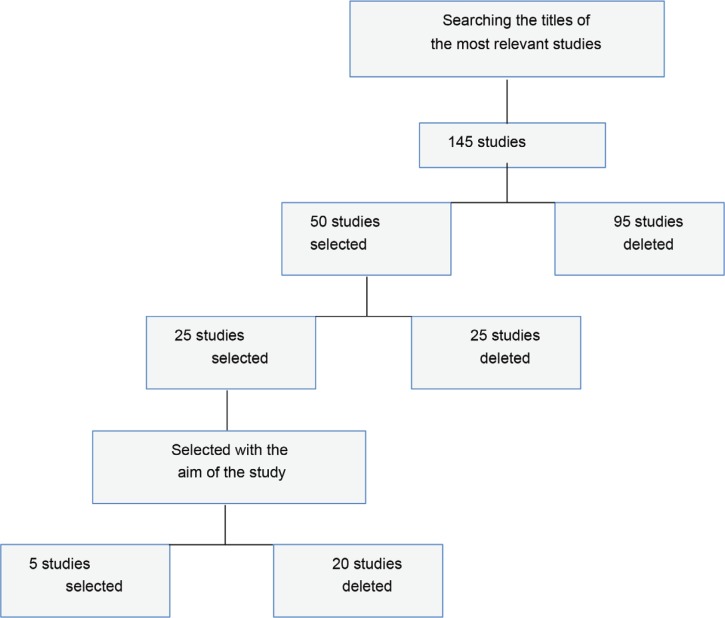
Flow chart of study selection process

The following databases were systematically searched between years 2015 to 2019 without any language restrictions in PUBMED, EMBASE, Web of Science, and Cochrane Library. In addition, the relevant references in the list of all included articles were also checked. The search term included “breast cancer”, “breast reconstruction” and “mastectomy”. In the preliminary study, 145 studies were selected which were selected by studying the titles of the most relevant studies (n=50). Fifty studies were conducted, and studies were conducted with the aim of the study (n=25). Then, the full text of 25 studies was studied. Finally, five studies that were included in the study were selected and evaluated (Figure 1).

*Study Inclusion and Exclusion Criteria*

Inclusion criteria were involving retrospective or prospective studies, patients with a diagnosis of breast cancer, and breast reconstruction after mastectomy. Exclusion criteria were involving reviews without original data, meta-analysis, case reports, and studies lacking control group.

*Data Extraction*

We recorded the following information for each study according to a prespecified protocol: author, year, study demographics, number and characteristics of participants, duration of follow-up, end-point data.

*Statistical Analysis*

We analyzed the data by the stata 14 software that was expressed risk ratio (RR) with 95% confidence interval (CI). 

## Results

**Fig. 2:**
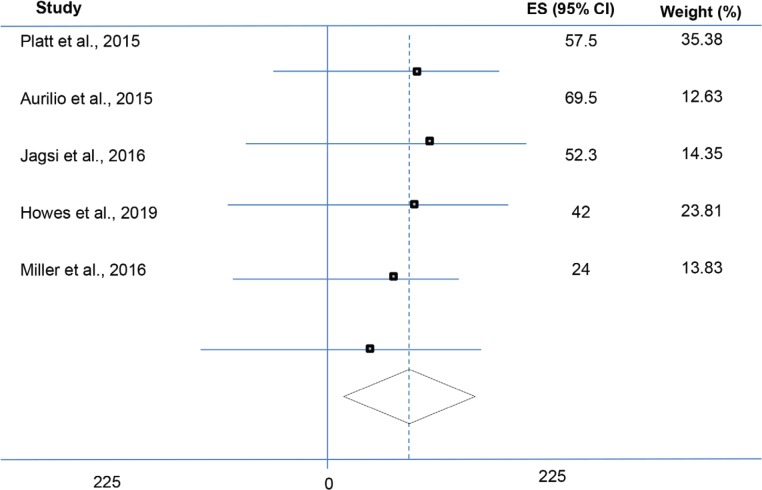
Forest plot of overall survival

The sample size was a range from 339 to 5644 patients. The median age was 46.3 years. Table 1 shows the data recorded and documented. A total of five studies reported data on the overall survival of advanced breast cancer patients and two studies reported data on the disease-free survival. There was no statistically significant heterogeneity between studies (Tables 2 and 3). The comprehensive therapy for breast cancer significantly changed over the last decade, and would clearly have an impact on survival and disease recurrence. The increased screening and means for detection of early disease and identification of patients with BRCA genes and genetic predispositions have also changed which would also impact survival and recurrence. Therefore, studies published after 2015 were assessed, and the results did not show any significant differences in terms of overall survival between two groups. Figure 2 showed Forest plot of overall survival.

**Table 1:** The findings and results of the studies that entered the study

**Result of study**		**Apparent survival benefit**	**Irrespective of the adjuvant treatment **	**Complication risks after immediate BR**	**Mastectomy and BR for cancer **	**Implant-based reconstruction compared to mastectomy **
Outcomes		Overall survival	Overall survival	Overall survival	Overall survival	Disease-free survival, overall survival
Follow up median	Mastectomy alone	23.4	8.6	Not available	Not available	Not available
	IBR	23.4	8.6	Not available	Not available	Not available
Lymph-node	Mastectomy alone	324/267/167	41/16/48	Not available	Not available	11, 5, 27
	IBR	384/236/138	137/102/100	Not available	21/37/22	5, 10,4
stage (I/II/III/I V)	Mastectomy alone	Not available	Not available	Not available	Not available	38, 19,28
	IBR	Not available	Not available	Not available	Not available	41,31,15
Age median	Mastectomy alone	45	Not available	Not available	Not available	58
	IBR	45	Not available	Not available	46	48
Patients (n)	Mastectomy alone	758	105	9250		210
	IBR	758	339	5644	400	681
Study design		RCS	CC	RCS	CC	PCR
Author		Platt et al., 2015	Aurilio et al., 2015	Jagsi et al., 2016	Howes et al., 2019	Miller et al., 2016

**Table 2:** Overall survival in to groups (without reconstruction and breast reconstruction)

**Author**	**Overall survival (without reconstruction)**	**Overall survival** **(breast reconstruction)**
Platt et al., 2015	57.5	47.5
Aurilio et al., 2015	69.5	79.5
Jagsi et al., 2016	52.3	74.6
Howes et al., 2019	42	57.9
Miller et al., 2016	24	76

**Table 3:** Heterogeneity Chi-Square

**Author**	**ES**	**95% Confidence Interval**		**Weight (%)**
		**Upper**	**Lower**	
Platt et al., 2015	57.5	-35.59	150.59	35.38
Aurilio et al., 2015	69.5	-86.31	225.31	12.63
Jagsi et al., 2016	52.3	-93.91	198.51	14.35
Howes et al., 2019	42.0	071.48	155.48	23.81
Miller et al., 2016	24	-124.95	172.95	13.83

I-squared (variation in ES attributable to heterogeneity)=0.0% Test of ES=0, z=1.77, P=0.077

## DISCUSSION

The present meta-analysis indicated that patients treated with breast reconstruction after mastectomy in women with breast cancer were comparable in terms of overall survival. Breast reconstruction is an option for women who are treated with mastectomy; however, there has been concern regarding the oncologic safety of BR. Two studies design were retrospective cohort study.^12^^,^^10^ Two studies design were case control study,^13^^,^^14^ and one study was prospective cohort study.^15^

Patients who received immediate breast reconstruction or mastectomy alone followed by PMRT, except in patients who were younger than 50 years of age. These younger patients appeared to have a survival advantage after immediate breast reconstruction. The survival outcomes are in relation to immediate breast reconstruction followed by PMRT for both the physicians and the patients.^18^ There are comparable survival outcomes compared to mastectomy alone, immediate post mastectomy reconstruction had limited advantage in survival after adjusting for confounding factor of family income. LABC patients who received immediate breast reconstruction or mastectomy alone followed by PMRT, except in patients who were younger than 50 years of age. These younger patients appeared to have a survival advantage after immediate breast reconstruction.^19^^,^^20^

Compared to mastectomy alone, immediate post mastectomy reconstruction had limited advantage in survival after adjusting for confounding factor of family income, if validated in other large databases, may help to illustrate the actual effect of immediate post mastectomy reconstruction on patient survival,^21^^,^^22^ It was shown that patients for whom implant-based reconstruction is available, immediate implant reconstruction does not increase the risk of lymphedema compared to mastectomy alone.^15^ It was demonstrated that women who undergo total mastectomy and breast reconstruction for cancer achieve a quality-of-life outcome that is at least as good as that following breast-conserving surgery.^14^

Furthermore, breast conservation has been found to be associated with lower physical well-being (i.e., more pain and discomfort) in the chest area and poorer sexual well-being outcomes.^23^^,^^24^ Overall survival, disease- free survival, and surgical site infection in patients who underwent IBR after mastectomy versus mastectomy alone, and the results demonstrated that there were no significant differences between IBR after mastectomy and mastectomy alone in overall survival, disease-free survival and local recurrence.^25^

The results revealed that patients who underwent IBR after mastectomy were associated with a significantly higher risk of surgical site infection. IBR patients exhibited a better survival trend that was maintained along a prolonged follow-up time, as exemplified by the survival curves.^26^ Also, no IBR group was characterized by unfavorable prognostic factors, including a higher number of metastatic lymph nodes. Taken together, the clinical data in the neoadjuvant and adjuvant setting favor the attitude to perform an IBR intervention, and not confirm preclinical evidence that points toward an augmented risk of relapse linked to breast reconstruction. IBR after mastectomy does not affect the overall survival and disease-free survival of breast cancer, which is in line with our findings.^13^

In either meta-analysis demonstrated no evidence for increased frequency of local breast cancer recurrence with IBR compared with mastectomy.^27^ At other systematic review study, rates of breast reconstruction were highly variable. Reconstruction appeared to be offered to a minority of women; around half took up the offer. The main reasons reported for no reconstruction included patient-related and adjuvant therapy-related factors. Clinicians’ beliefs about reconstruction may be an important factor. Rates of reconstruction could be increased with early discussion of the options when mastectomy is chosen or required.^28^^-^^30^

At a systematic review study of the literature, it was shown that IBR did not necessarily delay the start of adjuvant chemotherapy to a clinically relevant extent, suggesting that in general IBR was a valid option for non- metastatic breast cancer patients.^25^ We can conclude that IBR after mastectomy does not affect the overall survival and the incidence of surgical site infection in the IBR after mastectomy is higher than that in the mastectomy alone. The limitations of this study were failure to check disease-free survival, and incidence of local recurrence. It is recommended that these cases to be considered in future studies.
